# Thixotropy and Rheopexy of Muscle Fibers Probed Using Sinusoidal Oscillations

**DOI:** 10.1371/journal.pone.0121726

**Published:** 2015-04-16

**Authors:** David Altman, Fabio C. Minozzo, Dilson E. Rassier

**Affiliations:** 1 Department of Physics, Willamette University, Salem, Oregon, United States of America; 2 Department of Kinesiology and Physical Education, McGill University, Montreal, Quebec, Canada; 3 Departments of Kinesiology and Physical Education, Physiology, and Physics, McGill University, Montreal, Quebec, Canada; Semmelweis University, HUNGARY

## Abstract

Length changes of muscle fibers have previously been shown to result in a temporary reduction in fiber stiffness that is referred to as thixotropy. Understanding the mechanism of this thixotropy is important to our understanding of muscle function since there are many instances in which muscle is subjected to repeated patterns of lengthening and shortening. By applying sinusoidal length changes to one end of single permeabilized muscle fibers and measuring the force response at the opposite end, we studied the history-dependent stiffness of both relaxed and activated muscle fibers. For length change oscillations greater than 1 Hz, we observed thixotropic behavior of activated fibers. Treatment of these fibers with EDTA and blebbistatin, which inhibits myosin-actin interactions, quashed this effect, suggesting that the mechanism of muscle fiber thixotropy is cross-bridge dependent. We modeled a half-sarcomere experiencing sinusoidal length changes, and our simulations suggest that thixotropy could arise from force-dependent cross-bridge kinetics. Surprisingly, we also observed that, for length change oscillations less than 1 Hz, the muscle fiber exhibited rheopexy. In other words, the stiffness of the fiber increased in response to the length changes. Blebbistatin and EDTA did not disrupt the rheopectic behavior, suggesting that a non-cross-bridge mechanism contributes to this phenomenon.

## Introduction

Certain non-Newtonian fluids exhibit a time-dependent change in viscosity when they experience shear stress. When the viscosity of the fluid is reduced by shear stress, the material is said to exhibit thixotropy, and when shear stress increases the fluid’s viscosity, it is said to exhibit rheopexy [1]. Both phenomena typically result from reversible changes in microstructure. For example, for a thixotropic substance, weak interactions between polymers that make up the fluid are broken by agitation of the system, and these interactions re-form when agitation stops.

Buchtal and Kaiser [2] first used the term thixotropy to describe their observations that stretching an amphibian skeletal muscle fiber results in reduced fiber stiffness, and that the stiffness returns to its unperturbed value after a sufficient resting period. They described muscle thixotropy as resulting from disruption and re-formation of “entanglements” within the fibers.

Though the reason that muscle stiffness exhibits a history-dependence is not fully understood, it has been suggested that muscle thixotropy has important functional implications, including postural stability. According to this hypothesis, muscle’s increased stiffness in relaxed muscles helps maintain stability against unexpected external forces. When the muscle is activated and this stability is no longer required, the stiffness is reduced through thixotropic effects [3]. An understanding of muscle thixotropy is also important to our understanding of muscle function since there are many instances in which muscle is subjected to repeated patterns of lengthening and shortening. For example, muscles in the limbs undergo cyclic length changes during locomotion, and the muscle walls of a beating heart continually stretch and shorten [4].

In this report, we describe studies in which the mechanical properties of individual permeabilized muscle fibers were probed by applying sinusoidal length changes at one end of the fiber and measuring the resulting force at the other end. At frequencies above 1 Hz, this oscillation resulted in a history-dependent reduction in muscle fiber stiffness in activated muscle fibers. Inhibiting acto-myosin interactions caused this thixotropic effect to disappear, suggesting that the “entanglements” corresponding to this thixotropic effect are due to cross-bridge interactions. Surprisingly, at frequencies less than 1 Hz, oscillation resulted in a history-dependent *increase* in muscle fiber stiffness in activated fibers. Disruption of acto-myosin interactions has no effect on this rheopectic behavior, suggesting that there is a different mechanism of entanglement at play.

## Materials and Methods

### Solutions

Rigor solution (pH 7.0) was composed of (in mM) 50 Tris, 100 NaCl, 2 KCl, 2 MgCl_2_, and 10 EGTA. Dissection solution used for muscle storage and dissection (pH 7.0) was composed of (in mM) 20 imidazole, 100 KCl, 2 EGTA, 4 ATP and 7 MgCl_2_. Experimental solutions with pCa^2+^ of 4.5 (activating solution) and 9.0 (relaxing solution) (pH 7.0) were composed of (in mM) 20 imidazole, 14.5 creatine phosphate, 7 EGTA, 4 MgATP, 1 free Mg^2+^, free Ca^2+^ of 1 nM (relaxing) or 32 μM (activating), and KCl to adjust the ionic strength to 180. A pre-activation solution (pH 7.0, pCa^2+^ 9.0) was used prior to activating fibers, containing (in mM) 68 KCl, 0.5 EGTA, 20 imidazole, 14.5 PCr, 4.83 ATP, 0.00137 CaCl_2_, 5.41 MgCl_2_ and 6.5 HDTA.

Solutions containing blebbistatin were prepared according to procedures previously used in our laboratory [5]. Blebbistatin (Sigma, USA) was dissolved in dimethylformamide (DMF) to reach a concentration of 20 mM and was stored at −20°C before use. On the day of the experiment, 1 μL of the blebbistatin stock was diluted in 4 mL of activating (pCa^2+^ 4.5) or relaxing (pCa^2+^ 9.0) solutions to reach a final concentration of 5 μM. Care was taken to limit blebbistatin exposure to light, as it loses its effectiveness in wavelengths between 365 nm and 490 nm [6]. A red filter (650 nm) placed on the light source of the microscope was used to avoid exposure when the use of light was necessary during the experiments.

### Muscle fiber preparation

Small muscle bundles of the rabbit psoas were dissected, tied to wood sticks, and chemically permeabilized using rigor:glycerol (50:50) solutions following standard procedures [5, 7]. Fibers were stored at −20**°**C in rigor:glycerol solution.

On the day of the experiment, a muscle sample was transferred to a fresh rigor solution and stored in the fridge for one hour before use. A small section of the sample was extracted (~4 mm in length), and single fibers were dissected in dissection solution. The fibers were fixed at their ends with T-shaped clips made of aluminum foil, and were transferred to a temperature controlled experimental chamber to be attached between a force transducer (Model 400A, Aurora Scientific, Toronto, Canada) and a length controller (Model 312B, Aurora Scientific, Toronto, Canada). The protocol was approved by the McGill University Animal Care Committee and complied with the guidelines of the Canadian Council on Animal Care.

### Experimental protocol

After the fibers were set in the experimental chamber, which was kept at 5**°**C throughout the experiment, the average sarcomere length (SL) was calculated in relaxing solution using a high-speed video system (HVSL, Aurora Scientific 901A, Toronto, Canada) attached to a microscope. Images from a selected region of the fibers were collected at 1000–1500 frames/s, and the SL was calculated by fast Fourier transform (FFT) analysis based on the striation spacing produced by dark and light bands of myosin and actin, respectively. The length of the fiber was adjusted until the SL was 2.5 μm, which is along the plateau of the force-length relation for these fibers [8].

The fiber diameter and length were then measured using a CCD camera (Go-3, QImaging, USA; pixel size: 3.2 μm × 3.2 μm), and the cross-sectional area was estimated assuming circular symmetry.

We measured the response of muscle fibers to sinusoidal vibrations directed along the length of the fiber. Using the length controller, driving sinusoidal oscillations were applied for 20 seconds to the muscle fiber at frequencies ranging from 0.25 Hz to 30 Hz, and the force transducer was used to measure the resulting force at the opposite end of the fiber. The fiber was studied both in pCa^2+^ 9.0 (relaxed) and pCa^2+^ 4.5 (activated). For relaxed fibers, the magnitude of the oscillation was 2–3% of the fiber length, and for activated fibers, the magnitude of the oscillation was 1–1.5% of the fiber length. Throughout an experiment, we verified that fibers were not damaged by examining the fiber for consistency of the sarcomere pattern using the high-speed video system.

### Treatment of the fiber with EDTA and blebbistatin

Additional experiments were conducted following treatment of the fibers with EDTA (5 mM) and blebbistatin (5 μM). EDTA causes TnC extraction from myofibrils [9], and blebbistatin causes disruption of the cross-bridge cycle by binding to a site of the myosin S1 located between the nucleotide pocket and the cleft of the actin-binding site; this biases myosin into a pre-powerstroke conformation without directly affecting ATPase kinetics [10–12]. Together, both chemicals inhibit cross-bridge formations.

To conduct these experiments, oscillation data were first collected for the relaxed fiber prior to treatment with EDTA and blebbistatin. The fiber was then activated, and oscillation data were again collected. The fiber was then placed in rigor solution containing EDTA, the temperature was raised to 8°C, and the fiber was incubated for one hour. The temperature was then returned to 5°C, and the fiber was incubated in relaxing solution containing blebbistatin for 30 minutes. Oscillation data were collected in relaxing solution containing blebbistatin, the fiber was placed in activating solution containing blebbistatin, and oscillation data were collected again.

### Mechanical properties of muscle fibers

When modeling a muscle fiber as a linear viscoelastic element, we approximated the final five oscillations of a 20-second force time-trace (by which time the oscillation had reached equilibrium) using the relation

$$F=F_{o}+F_{1}\sin(2\pi ft+\delta_{1})$$ (1)

where *F*_*o*_ is the mean force, *f* is the frequency of the driving oscillation, and *F*_*1*_ and *δ*_1_ are the amplitude and phase of the first harmonic, as determined by Fourier analysis. The length time trace was described by a similar relation. The phase of the length time trace was set to zero, and thus *δ*_1_ describes the phase difference between the length and force time traces.

We described the viscoelastic properties of the fiber using the framework described by Kawai et al. [13]. The complex modulus describes both the viscous and elastic properties of a material, and is described by the ratio of the tension change (force divided by the cross-sectional area) and the fractional length change (change in length divided by the fiber length). The magnitude of the complex modulus was calculated at each frequency according to the relation

$$\left|Y(f)\right|=\frac{F_{1}(f)}{L_{1}(f)}\times \frac{L_{o}}{A_{o}}$$ (2)

where *L*_*o*_ is the length of the muscle fiber and *A*_*o*_ is its cross-sectional area. Cylindrical shape was assumed in order to calculate the area.

From the magnitude of the complex modulus and the phase, we calculated the elastic modulus (*G*) and viscous modulus (*R*) through the relations

$$\begin{array}{l} G(f)=\left|Y(f)\right|\cos\delta_{1}\\ R(f)=\left|Y(f)\right|\sin\delta_{1} \end{array}$$ (3)

## Results

**Fig 1:**
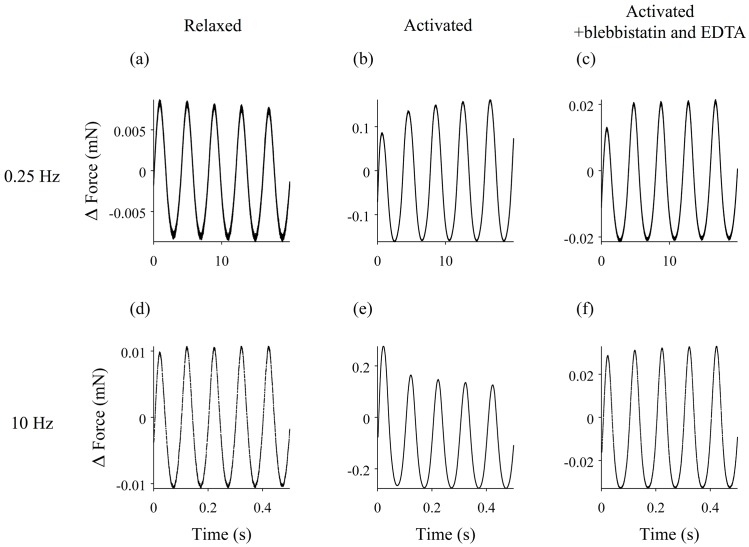
Length change and force response of a muscle fiber experiencing driving sinusoidal oscillations. Length changes are shown in black, and the force response is in red. The mean force and length have been subtracted from the traces. 0.25 Hz oscillations of (a) the relaxed fiber, (b) the activated fiber, and (c) the activated fiber following treatment with EDTA and blebbistatin. 10 Hz oscillation of (d) the relaxed fiber, (e) the activated fiber, and (f) the activated fiber following treatment with EDTA and blebbistatin. The length of the fiber was 2.6 mm.

Individual muscle fibers were subjected to sinusoidal length changes at one end at frequencies ranging from 0.25 to 30 Hz, and the resulting force response was measured at the other end of the fiber. Examples of length and force time traces at different experimental conditions are shown in Fig 1.

Such data can also be represented as Lissajous figures in which the force response is plotted as a function of length (S1, S2, and S3 Figs). To highlight changes in the force response over time, these figures show both the initial driven oscillation (red) and the final driven oscillation (black) after twenty seconds of oscillation.

**Fig 2:**
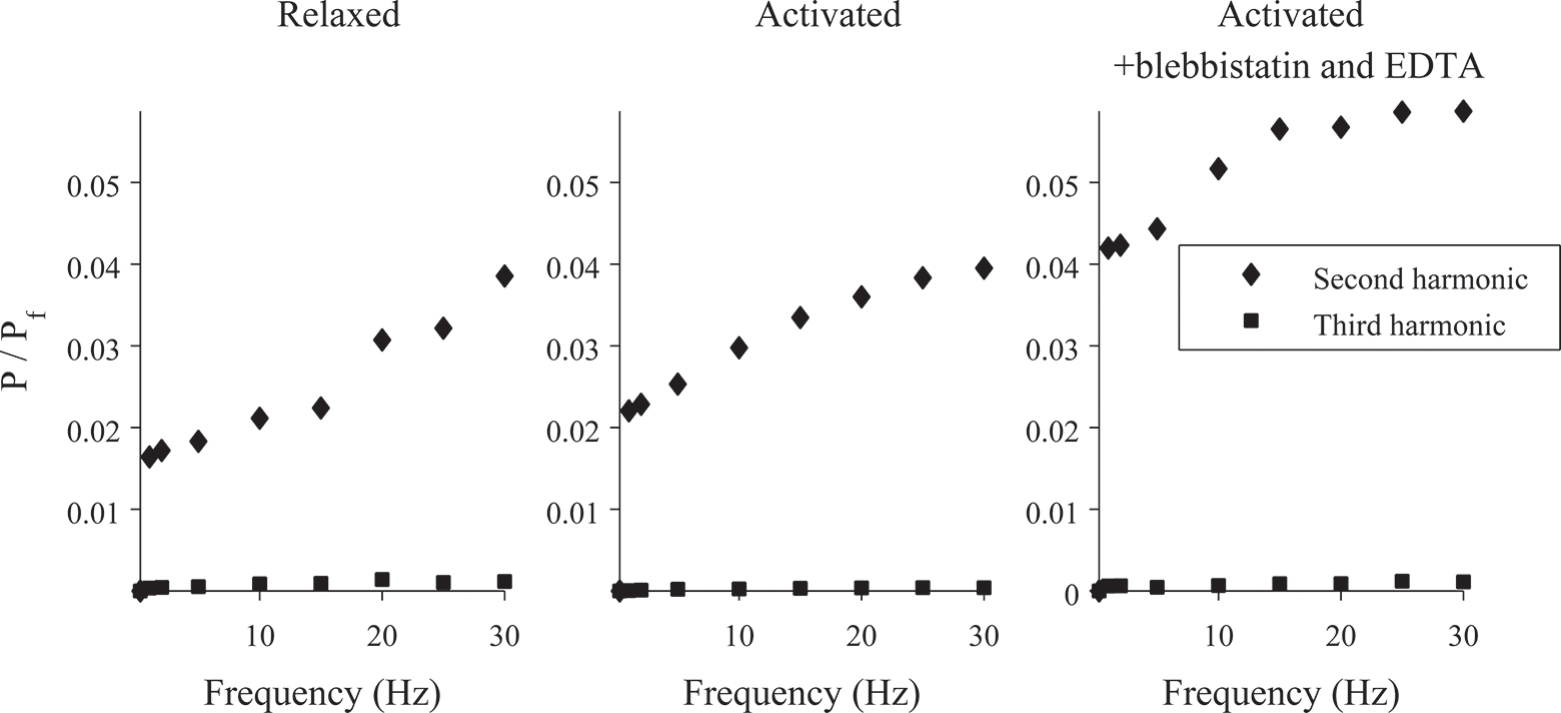
Frequency components of the force signal for a muscle fiber experiencing sinusoidal oscillations. A Fourier transform was applied to the force measurement time-courses, and the powers of the signal at the fundamental frequency (which is the same as the driving frequency) and the next two harmonics were calculated. Plotted are the ratios of the power at the second or third harmonic (*P*) and the power at the fundamental frequency (*Pf*) for three different experimental conditions.

If the muscle fiber were a linear viscoelastic material, the Lissajous figures would be ellipses. As can be seen, the plots are not ellipses, indicating a nonlinearity that becomes more pronounced at higher frequencies of oscillation. To characterize this, a Fourier transform was applied to the force time-traces, and the powers of the signal at the fundamental frequency (which is the same as the frequency of the length changes) and the next two harmonics were calculated. An example of this analysis is shown in Fig 2, where we have plotted the ratios of the power at the second or third harmonic (*P*) and the power at the fundamental frequency (*P*_*f*_) for various experimental conditions. As can be seen, the non-linearity in the signal primarily arises from the second harmonic, and this component becomes larger as the driving frequency increases.

For driven oscillations in which the non-linearity was relatively small, the muscle fiber was modeled as a linear viscoelastic element. Specifically, the fiber was modeled as linear when the power of the signal at the second harmonic was less than or equal to 2.5% of the power at the fundamental frequency for a relaxed muscle fiber, which corresponded to frequencies of 15 Hz or less (Fig 2, left). It is worth noting, however, that the nonlinearity is more pronounced in activated fibers (Fig 2, middle and right). For example, for an activated fiber driven at 15 Hz, the power of the signal at the second harmonic is ~3% of the power at the fundamental.

### Analysis of relaxed fibers

**Fig 3:**
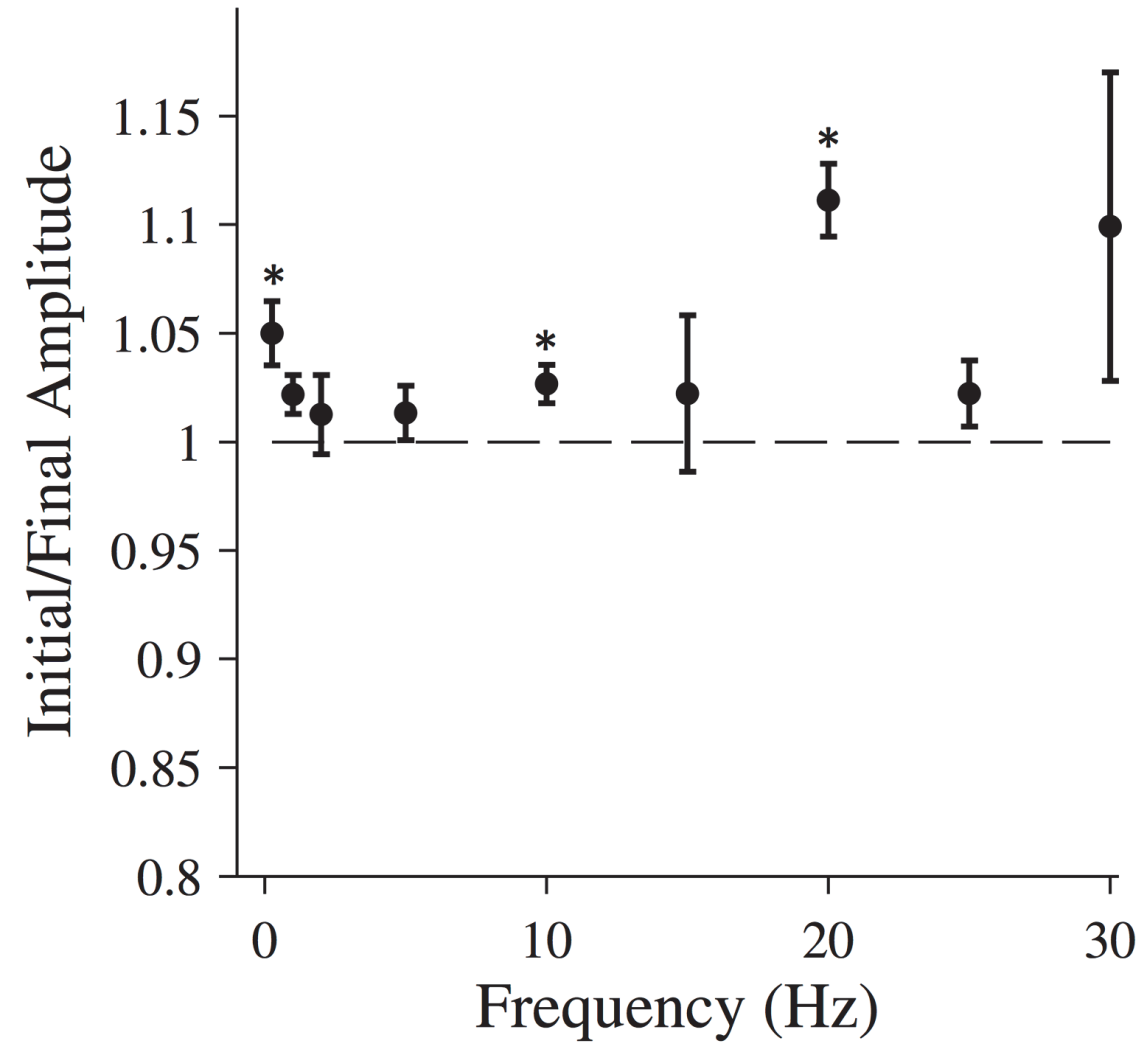
Changing force response for relaxed muscle fibers. The ratio of the initial and final RMS amplitudes for relaxed muscle fibers. The total length of the oscillation was 20 seconds. All data points are (MEAN±SEM), and the number of fibers analyzed was N = 6. A one-sample t-test was used to test the null hypothesis that data collected at each frequency comes from a normal distribution with mean equal to 1. For data points marked with an asterisk, the null hypothesis was rejected (p<0.05).

The force response of relaxed fibers does not appear to change significantly over time (Fig 1). To quantify this behavior, we calculated the root-mean-squared (RMS) amplitude of both the first force oscillation and the final force oscillation after twenty seconds. The ratio of these two values is plotted as a function of driving frequency in Fig 3. While these data suggest that there may be a slight decrease in the stiffness of the fiber over time, the ratio is statistically indistinguishable from 1 at most driving frequencies, indicating that the amplitude is similar at the beginning and end of the driving oscillation.

**Fig 4:**
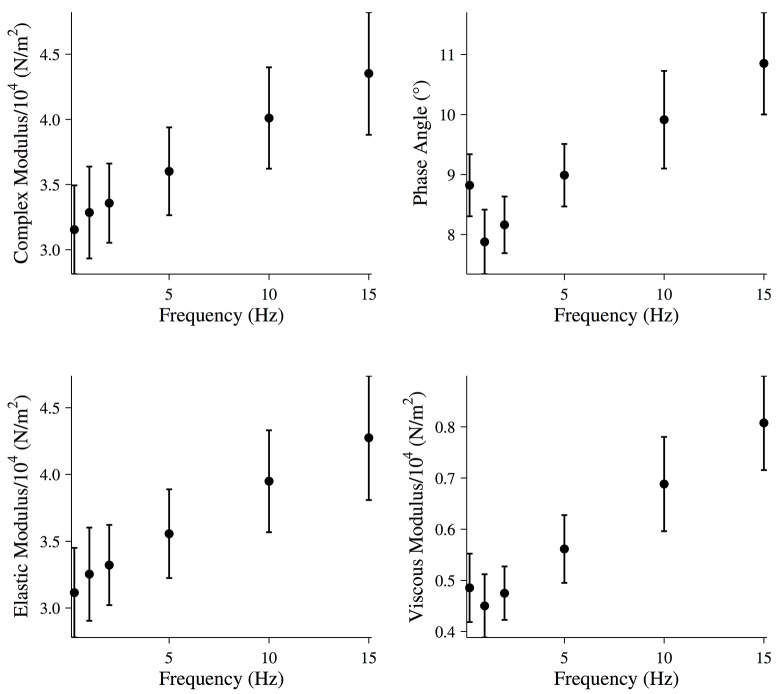
Mechanical properties of relaxed muscle fibers. Magnitude of the complex modulus, phase, elastic modulus, and viscous modulus for relaxed muscle fibers during the final five oscillations of the driven oscillation. All data points are (MEAN±SEM), and the number of fibers analyzed was N = 6.

For frequencies of 15 Hz or less, the mechanical properties of the fiber were calculated assuming the fiber is a linear viscoelastic material. The magnitude of the complex modulus and the viscous and elastic moduli of the fiber were calculated at each frequency as described in the Materials and Methods (Fig 4).

### Analysis of activated fibers

Upon activation, muscle fibers became stiffer, as evidenced by a considerably larger force response despite a smaller amplitude length change. At nearly all oscillation frequencies, the force response changed during the 20-second oscillation (Fig 1). For a driving frequency of 0.25 Hz, the fiber became stiffer over time, resulting in an increase in the amplitude of the force response (Fig 1B). At 10 Hz, the opposite was true; over time, the amplitude of the force response decreased, indicating that the fiber was becoming softer (Fig 1E).

**Fig 5:**
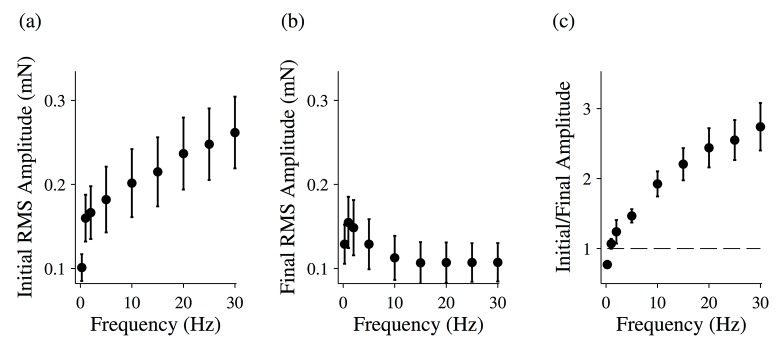
Changing force response for activated muscle fibers. The root-mean-squared (RMS) amplitude for the initial driving oscillation (a) and the final driving oscillation (b) for activated muscle fibers. (c) The ratio of the initial and final RMS amplitudes for activated muscle fibers. The total length of the oscillation was 20 seconds. All data points are (MEAN±SEM), and the number of fibers analyzed was N = 8.

To quantify this effect, we again calculated the RMS amplitude of each force time trace’s first oscillation (Fig 5A) and last oscillation (Fig 5B), and we calculated the ratio of these amplitudes (Fig 5C). Below 1 Hz, this ratio is less than 1, indicating the fiber is growing stiffer over time. Above 1 Hz, the ratio is greater than 1, indicating that the fiber is softening. Furthermore, the ratio gets larger as the driving frequency is increased.

It is worth noting that, for frequencies of 10 Hz or greater, the final RMS amplitude is the same for all frequencies (Fig 5B). In other words, the increase seen in the ratio plotted in Fig 5C results not from an increased softening of the fiber with frequency, but rather results from an increased initial stiffness at higher frequencies.

**Fig 6:**
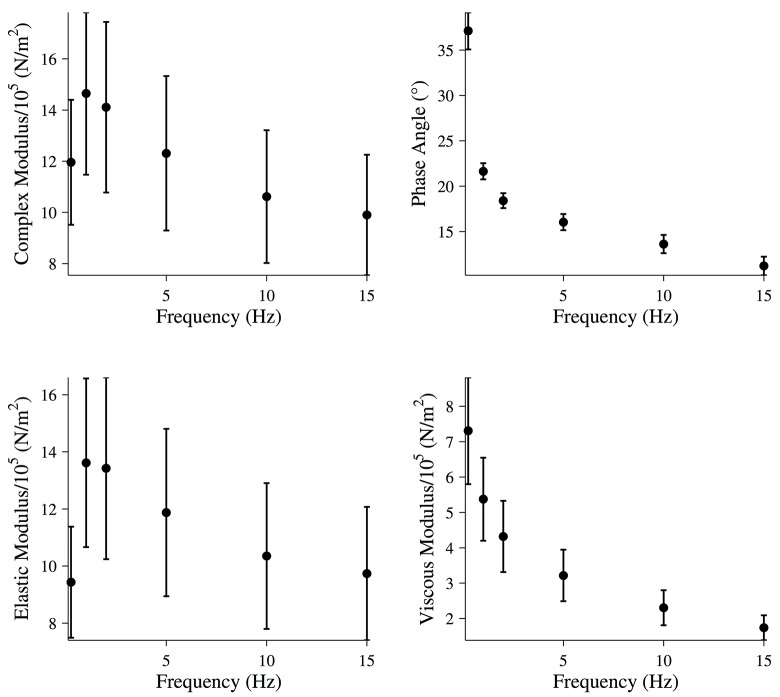
Mechanical properties of activated muscle fibers. Magnitude of the complex modulus, phase, elastic modulus, and viscous modulus for activated muscle fibers during the final five oscillations of the driven oscillation. All data points are (MEAN±SEM), and the number of fibers analyzed was N = 8.

For driving oscillations of 15 Hz or less, the last five oscillations of the force response were used to calculate the magnitude of the complex modulus and the viscous and elastic moduli of the fiber (Fig 6). Although there was a large variability in the magnitude of these values, the fibers exhibited the same general pattern. The response of the fiber was maximal at 1 Hz, where the elastic modulus of the fiber peaked, while the viscous modulus decreased with increasing frequency.

### Analysis of activated fibers treated with EDTA and blebbistatin

The experiments described above were repeated following treatment of the muscle fiber with EDTA and blebbistatin to disrupt acto-myosin interactions. As with untreated relaxed fibers, the amplitude of the force response remained fairly constant during the duration of the driving oscillation (S4 Fig), although the viscoelastic properties were somewhat changed (S5 Fig).

**Fig 7:**
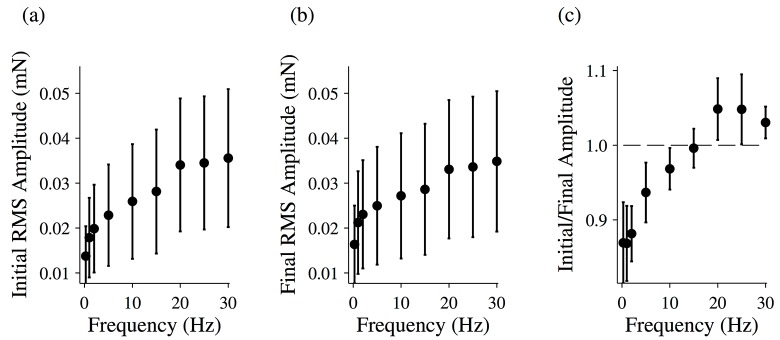
Changing force response for activated muscle fibers following treatment. The root-mean-squared (RMS) amplitude for the initial driving oscillation (a) and the final driving oscillation (b) for activated muscle fibers following treatment with EDTA and blebbistatin. (c) The ratio of the initial and final RMS amplitudes for activated muscle fibers following treatment with EDTA and blebbistatin. The total length of the oscillation was 20 seconds. All data points are (MEAN±SEM), and the number of fibers analyzed was N = 2.

Activated fibers, on the other hand, changed behavior dramatically following treatment. At all frequencies, the magnitude of the measured force time trace was decreased, indicating a reduction in the stiffness of the fiber. Rheopexy was still observed for low frequency driving oscillations (Fig 1C), but the thixotropy at higher driving frequencies disappeared (Fig 1F). Again, we calculated the RMS amplitude of each force time trace for the initial oscillation (Fig 7A) and the final oscillation (Fig 7B) and calculated the ratio of the two (Fig 7C). The ratio is less than one for low frequencies, while at higher frequencies the ratio is ~1.

**Fig 8:**
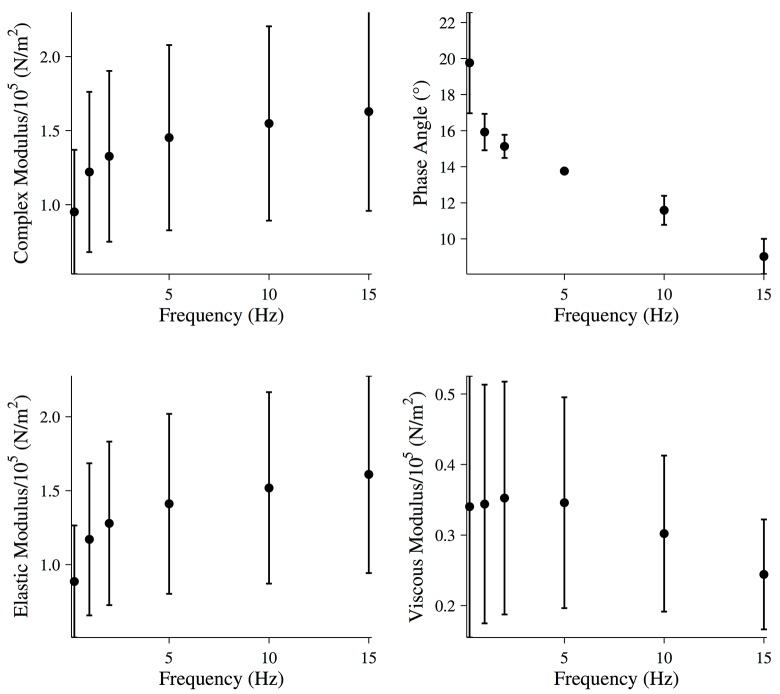
Mechanical properties of activated muscle fibers following treatment. Magnitude of the complex modulus, phase, elastic modulus, and viscous modulus for activated muscle fibers during the final five oscillations following treatment with EDTA and blebbistatin. All data points are (MEAN±SEM), and the number of fibers analyzed was N = 2.

For driving oscillations of 15 Hz or less, the last five oscillations of the force response were used to calculate the magnitude of the complex modulus and the viscous and elastic moduli of the fiber (Fig 8).

## Discussion

Muscle thixotropy refers to the apparent temporary reduction in muscle stiffness following movement. Intact fiber measurements of relaxed muscle fibers indicate that thixotropy in muscle arises from a history dependent, short-range elastic component (SREC) [14–16], although the nature of the SREC is still debated. One model posits that muscle thixotropy results from a temporary reduction in the number of attached cross-bridges following movement [15, 17, 18]. However, it has also been suggested that this history dependent behavior could result from titin filaments, whose mechanical properties change in response to force [19–22]. Arguments for and against each of these hypotheses have been discussed in the literature [23, 24].

To help differentiate between these models, we treated muscle fibers with EDTA and blebbistatin to specifically disrupt acto-myosin interactions. Although there is no evidence that blebbistatin treatment directly affects titin properties, it is possible that thin filament extraction may affect the overall sarcomere stiffness as titin binds to actin. However, we believe that such mechanism was not present in our study for two reasons. First, the passive forces produced at low Ca^2+^ concentration were similar before and after extraction of thin filaments. Second, it has been shown that the potential effects of Ca^2+^ on titin stiffness are independent of titin-actin interactions, and are associated only with stiffening of titin molecules [25, 26].

### Thixotropy

We studied the history-dependent stiffness of muscle fibers by measuring the force response of muscle fibers experiencing sinusoidal length changes. For driving frequencies greater than ~1 Hz, activated fibers exhibited thixotropy. There are similarities between experiments performed on permeabilized fibers at pCa^2+^ 9.0, presented in this paper and by other authors [4, 27], and experiments performed on relaxed intact fibers [15, 28, 29]. Hill showed that the response of resting intact skeletal muscles to ramp stretches is biphasic [15]. The force rises rapidly during the initial phase of the stretch, corresponding to the SREC described above. The SREC exhibits thixotropy qualitatively similar to what was observed in our study, suggesting that thixotropy is a general phenomenon encountered both in intact and permeabilized muscles fibers at different levels of activation.

Disruption of acto-myosin interactions resulted in quashed thixotropy of activated fibers, suggesting that this thixotropy arises primarily from a cross-bridge mechanism. We hypothesized that the cross-bridge dependent thixotropy at higher frequencies results from force-dependent cross-bridge kinetics. Specifically, we postulated that forces within a cross-bridge perturb mechanical transitions within the actomyosin chemomechanical cycle. This, in turn, affects the mechanical properties of the fiber by altering both the number and state of bound cross-bridges within a sarcomere. To test this model, we simulated the force response of a half-sarcomere experiencing a sinusoidal length oscillation.

#### Simulating the cross-bridge dependent thixotropy

**Fig 9:**
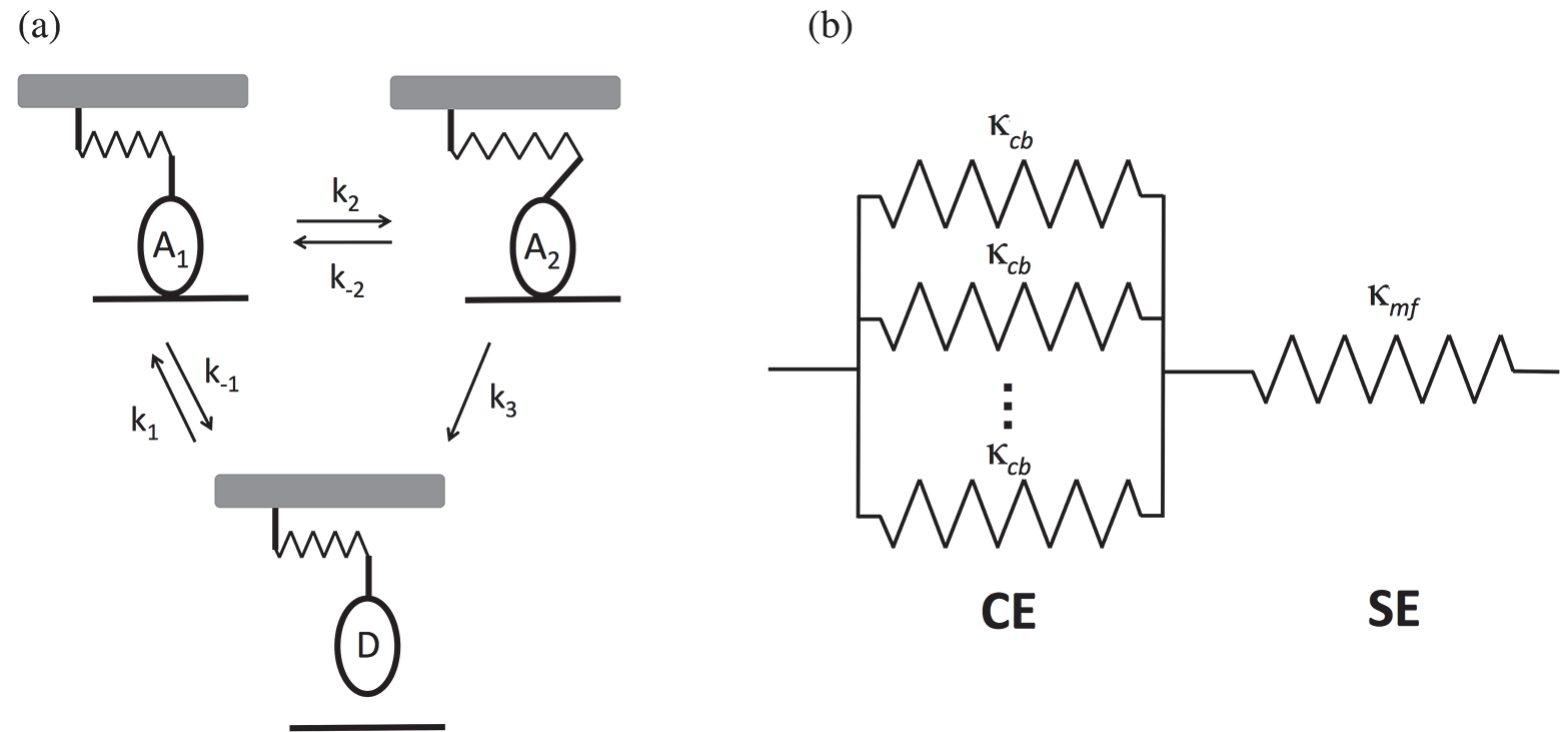
Model of the half-sarcomere. (a) Individual cross-bridges are linearly elastic elements that pass through three states: two attached states (A_1_ and A_2_) and a detached state (D). The transition from A_2_ to D is irreversible, and the transition from A_1_ to A_2_ corresponds to the powerstroke. (b) Model of the half-sarcomere. The half-sarcomere was modeled as a cross-bridge element (CE), consisting of cross-bridges attached in parallel, which is in series with the series element (SE), which represents the myofilament compliance.

A model of a half-sarcomere was developed that is based on the framework of Campbell and Moss [4, 27]. According to this model, a single myosin cycles through three-states: D (detached), A_1_ (first attached), and A_2_ (second attached) (Fig 9A). The transition between A_1_ and A_2_ corresponds to the powerstroke, and results in an increase in the length of the cross-bridge attachment of *x*_*ps*_. The cross-bridge is modeled as a linear elastic element with stiffness *κ*_*cb*_.

In our model, the four rate constants *k*_*-1*_, *k*_*2*_, *k*_*-2*_, and *k*_*3*_ depend on the force exerted on the cross bridge. We posit that the transition associated with a force-dependent rate constant involves a mechanical step, and that the force on the cross bridge perturbs this mechanical transition according to the relation described in [30]

$$k_{i}=k_{i}^{o}\exp\left(\frac{F_{cb}\delta_{i}}{k_{B}T}\right)$$ (4)

where $k_{i}^{o}$ is the value of the rate in the absence of any force on the cross-bridge, *F*_*cb*_ is the force on the cross-bridge, *δ*_*i*_ is the distance to the transition state of the mechanical transition, and *k*_*B*_ *T* is the Boltzmann constant times the temperature.

The force results from stretching of the elastic cross-bridge away from its equilibrium position. Thus, the force exerted by a cross-bridge along the direction of extension is given by

$$F_{cb}=-\kappa_{cb}l_{cb}$$ (5)

where *l*_*cb*_ is the length by which the cross-bridge has been extended (or for negative values, compressed). When the motor first binds to the filament, it is assumed that *l*_*cb*_ *= 0*. When the motor transitions from A_1_ to A_2_, a powerstroke occurs, which causes *l*_*cb*_ to increase by *x*_*ps*_. Similarly, *l*_*cb*_ decreases by *x*_*ps*_ during the transition from A_2_ to A_1_.

*δ*_2_ and *δ*_3_ are assumed to be positive, indicating that a positive extension of the cross-bridge increases the associated rates, while *δ*_−2_ and *δ*_−1_ are negative, indicating that these rates are slowed by a positive extension. Furthermore, since the transition between A_1_ and A_2_ corresponds to the powerstroke, the sum of *δ*_−2_ and *δ*_2_ is assumed to be equal to *x*_*ps*_.

We used this kinetic scheme to model the force-response of a half-sarcomere experiencing a length oscillation. We modeled the half-sarcomere as containing 294 cross-bridges [31], and we assumed that all the cross-bridges that are attached behave as linear elastic elements in parallel (Fig 9B). The resulting elastic element is referred to as the cross-bridge element (CE). In series with the CE is a linear elastic element (referred to as the series element (SE)) with stiffness *κ*_*mf*_, which corresponds to the compliance of the myofilament in the absence of cross-bridge attachments.

To model this system, we performed the following Monte Carlo simulation:

#### Initial conditions—Step 1

The initial state of the system is set with an equal number of motors in each of the three states A_1_, A_2_, and D. The displacement of each of the attached cross-bridges is set to zero, and so according to Equation (5) all cross-bridges experience no net force. The displacement of the series elastic element is also set to zero, and so this element also experiences no net force.

#### Equilibration—Step 2

For a single cross-bridge in the half-sarcomere, the rate constants are determined from Equations (4) and (5). The state of the cross-bridge after a small interval of time (set to Δ*t* = 10^−4^ s) is then determined as follows. If the initial state of the cross-bridge is state D, then it has two possible actions: it can move to state A_1_ or it can stay in state D. The probabilities for these events are given by

$$p_{D\to A_{1}}=k_{1}\Delta t$$ (6)

$$p_{D\to D}=1-p_{D\to A_{1}}$$ (7)

If the cross-bridge starts in state A_1_, it can either move to state A_2_ or D, or it can stay in state A_1_. The probabilities for each of these are given by

$$p_{A_{1}\to A_{2}}=k_{2}\Delta t$$ (8)

$$p_{A_{1}\to D}=k_{-1}\Delta t$$ (9)

$$p_{A_{1}\to A_{1}}=1-p_{A_{1}\to A_{2}}-p_{A_{1}\to D}$$ (10)

Finally, if the cross-bridge starts in state A_2_, it can either move to state A_1_ or D, or it can stay in state A_2_. The probabilities for each of these are given by

$$p_{A_{2}\to D}=k_{3}\Delta t$$ (11)

$$p_{A_{2}\to A_{1}}=k_{-2}\Delta t$$ (12)

$$p_{A_{2}\to A_{2}}=1-p_{A_{2}\to D}-p_{A_{2}\to A_{1}}$$ (13)

Using a random number generator and the relevant probabilities calculated above, the state of the motor after an interval of time Δ*t* is determined. If during this interval of time, the cross-bridge transitions between states A_1_ and A_2_, the value of the displacement *l*_*cb*_ is changed by ±*x*_*ps*_, depending on the direction of the transition.

#### Step 3

Step 2 is repeated for each cross-bridge in the half-sarcomere.

#### Step 4

The force exerted by each cross-bridge is re-calculated using Equation (5), and the forces from each cross-bridge are summed to yield the net-force exerted by the CE. At this point, the CE and SE are no longer in equilibrium (the forces they exert on each other are no longer equal), and so the point of contact between them is allowed to move to return the system to equilibrium. Specifically, this point moves by a distance

$$\Delta x=\left(F_{CE}^{f}-F_{CE}^{i}\right)/\kappa_{mf}$$ (14)

where $F_{CE}^{i}$ and $F_{CE}^{f}$ are the forces exerted on the CE before and after step (3), respectively. Because moving this point changes the displacement of each cross-bridge, Δ*x* is added to *l*_*cb*_ for each cross-bridge.

#### Step 5

Steps 2–4 are repeated until the sarcomere has evolved for 1 second. At this point, the system has reached equilibrium (data not shown).

#### Length oscillation—Step 6

The length change is a sinusoidal function given by the function

E=Asin(2πft) (15)

where *A* is the amplitude of the oscillation and *f* is its frequency. The change in length Δ*E* is calculated for the next interval of time Δ*t*. Δ*E* changes the displacement from equilibrium for both the CE and SE. Specifically, the CE is extended by

$$\Delta x_{oscillation}=\Delta E\frac{\kappa_{mf}}{\kappa_{mf}+N_{attached}\kappa_{cb}}$$ (16)

where *N*_*attached*_ is the total number of cross-bridges in states A_1_ or A_2_. Δ*x*_*oscillation*_ is added to the displacement *l*_*cb*_ for each cross-bridge.

#### Step 7

Steps 2–4 are repeated to determine the next state of each cross-bridge after another interval of time Δ*t*.

#### Step 8

Steps 6 and 7 are repeated until the sarcomere has evolved for 2 seconds in the presence of the oscillation.

**Fig 10:**
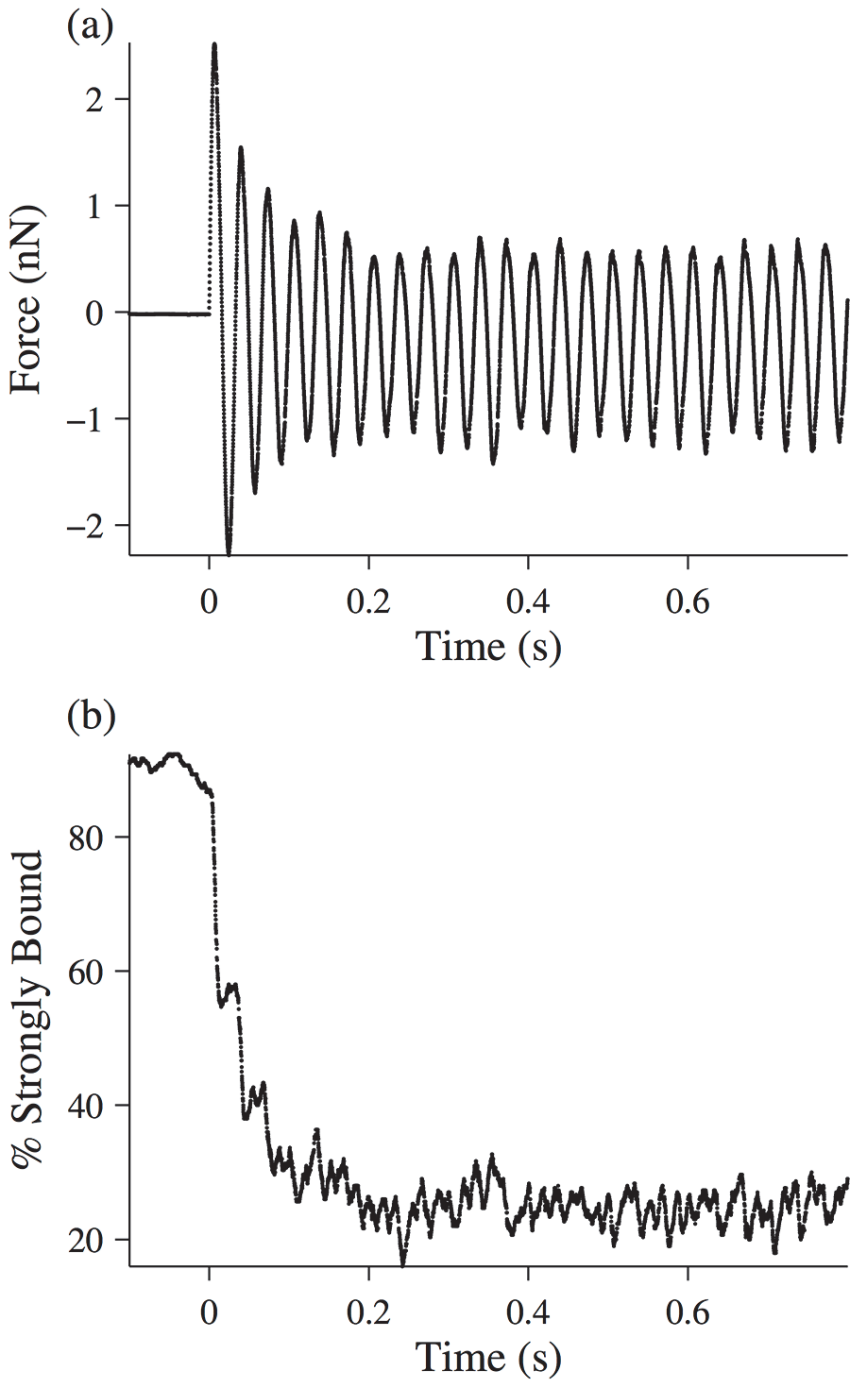
Simulation of a half-sarcomere experiencing a 30 Hz length oscillation. The simulation is described in detail in the text, and the parameters used are found in Table 1. (a) Force-response time-trace of the half-sarcomere. (b) Percent of strongly bound cross-bridges (i.e. in one of the attached states) as a function of time.

The simulation described above was run with the parameters in Table 1. The force response of the fibers was sinusoidal, and consistent with our hypothesis, the half-sarcomere exhibited thixotropy for frequencies of 5 Hz and higher (Fig 10A). Plotting the percent of strongly-bound cross-bridges as a function of time during the oscillation (Fig 10B) shows that this change in the mechanical properties of the fiber results from a change in the number of bound cross-bridges within a sarcomere

**Table 1:** Parameter values for the model of a half-sarcomere.

$k_{3}^{o}$	4 s^−1^	*k*_1_	10 s^−1^
*δ*_3_	1×10^−9^ m	$k_{-1}^{o}$	0.6 s^−1^
*A*	1.3×10^−7^ m	*δ*_−1_	−1×10^−9^ m
*κ*_*cb*_	1×10^−4^ N/m	$k_{2}^{o}$	1 s^−1^
*κ*_*mf*_	0.5 N/m	*δ*_2_	4×10^−9^ m
*x*_*ps*_	5×10^−9^ m	$k_{-2}^{o}$	1 s^−1^
T	293 K	*δ*_−2_	−1×10^−9^ m

Parameters used for the simulation of a half-sarcomere experiencing driving sinusoidal oscillations. The model is described in detail in the text.

**Fig 11:**
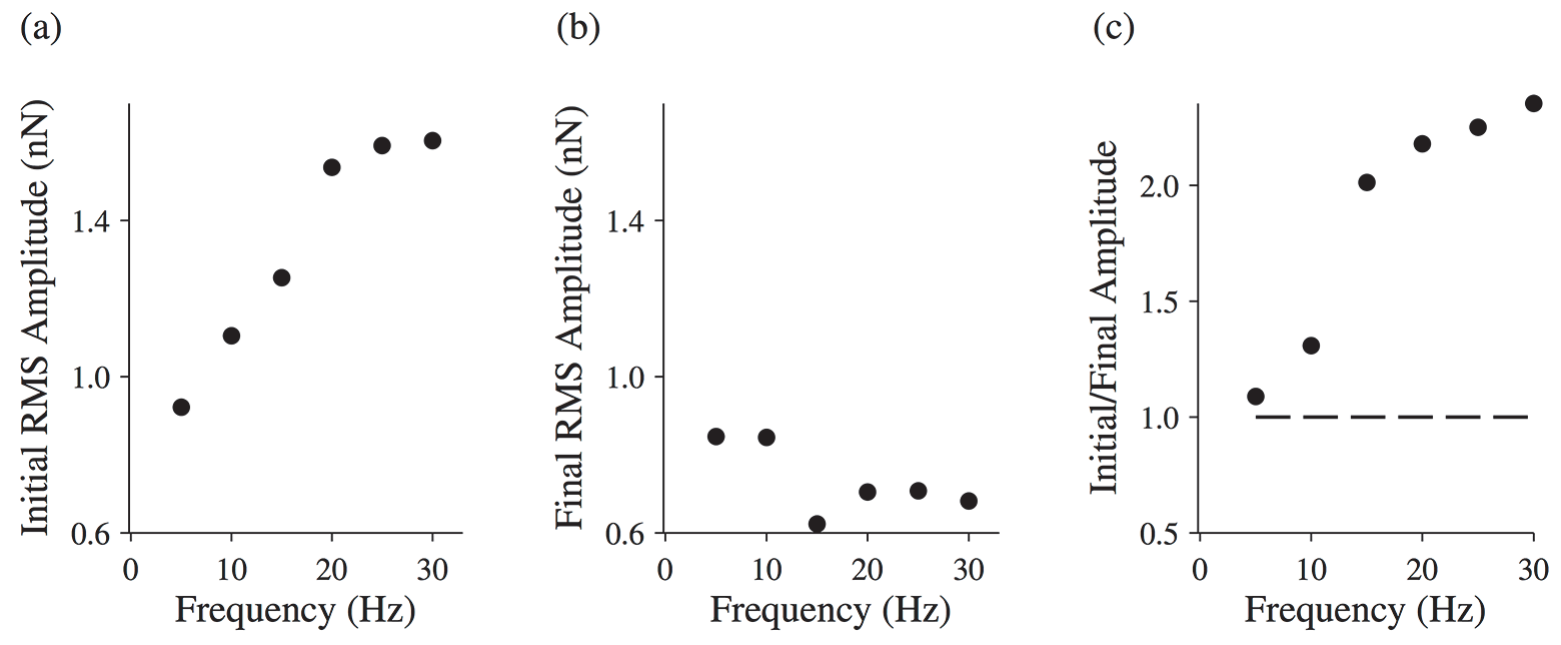
Changing force response for a simulated half-sarcomere. The root-mean-squared (RMS) amplitude for the initial driving oscillation (a) and the final driving oscillation (b) for a simulated half-sarcomere. (c) The ratio of the initial and final RMS amplitudes for a simulated half-sarcomere. The total length of the oscillation was 2 seconds.

In addition, we calculated the RMS amplitude of the first oscillation (Fig 11A) and last oscillation (Fig 11B) for each simulated force trace, and plotted the ratio of these amplitudes as a function of frequency (Fig 11C). Consistent with our data, the magnitude of the thixotropic effect was greater at higher frequencies. Also consistent with our data, the thixotropic effect arises primarily because of an increase in the initial RMS amplitude, with the final RMS amplitude remaining fairly constant as the frequency is varied.

The simulations were run with the temperature in Equation (4) set to 293 K (20**°**C). The simulation was also repeated at the temperature of the experiment (278 K) and at *in vivo* temperatures (310 K). Results were nearly indistinguishable (S6 Fig), suggesting that this thixotropic mechanism can account for thixotropy across a range of temperatures.

### Rheopexy

At frequencies less than 1 Hz, we observed a novel rheopectic behavior of activated muscle fibers. This rheopexy was still pronounced following treatment with blebbistatin and EDTA, suggesting an as of yet unknown mechanism that is not cross-bridge dependent.

It is worth noting that before treatment with blebbistatin and EDTA, rheopectic behavior was only seen at oscillation frequencies less than 1 Hz, but following treatment, it was observed at frequencies less than 20 Hz. A likely explanation is that, in the range between 1 Hz and 20 Hz, there are both rheopectic and thixotropic mechanisms at play. However, thixotropy is more pronounced and thus dominates the observed changes in stiffness of the fiber in this frequency range. When we quash the thixotropic effect with EDTA and blebbistatin, the rheopexy then becomes apparent at higher frequencies.

In summary, our results suggest that myosin cross-bridges are responsible for the thixotropic behavior of muscle fibers. Rheopectic behavior at lower frequencies, on the other hand, is caused by a cross-bridge independent mechanism.

## Supporting Information

S1 Fig Lissajous figures for a relaxed muscle fiber experiencing driving sinusoidal oscillations. The frequency of oscillation in Hz is indicated in the top left corner of the plot. The red line is the first oscillation and the black line is the final oscillation after 20 seconds. The x-axis and y-axis are is 0.1 mm and 0.03 mN in length, respectively. The length of the muscle fiber was 2.6 mm.
(TIFF)

S2 Fig Lissajous figures for an activated muscle fiber experiencing driving sinusoidal oscillations. The frequency of oscillation in Hz is indicated in the top left corner of the plot. The red line is the first oscillation and the black line is the final oscillation after 20 seconds. The x-axis and y-axis are is 0.05 mm and 0.8 mN in length, respectively. The length of the muscle fiber was 2.6 mm.
(TIFF)

S3 Fig Lissajous figures for an activated muscle fiber experiencing driving sinusoidal oscillations following treatment with EDTA and blebbistatin. The fiber is experiencing driving sinusoidal oscillations, and the frequency of oscillation in Hz is indicated in the top left corner of the plot. The red line is the first oscillation and the black line is the final oscillation after 20 seconds. The x-axis and y-axis are is 0.05 mm and 0.11 mN in length, respectively.
(TIFF)

S4 Fig Changing force response for relaxed muscle fibers following treatment. The ratio of the initial and final RMS amplitudes for relaxed muscle fibers following treatment with EDTA and blebbistatin. The total length of the oscillation was 20 seconds. All data points are (MEAN±SEM), and the number of fibers analyzed was N = 2. A one-sample t-test was used to test the null hypothesis that data collected at each frequency comes from a normal distribution with mean equal to 1. The null hypothesis could not be rejected for all data points (p>0.05).
(TIFF)

S5 Fig Mechanical properties of relaxed muscle fibers following treatment. Magnitude of the complex modulus, phase, elastic modulus, and viscous modulus for relaxed muscle fibers during the final five oscillations following treatment with EDTA and blebbistatin. All data points are (MEAN±SEM), and the number of fibers analyzed was N = 2.
(TIFF)

S6 Fig Simulated force-response of a half-sarcomere to a 30 Hz length oscillation. The simulation is described in detail in the text, and the parameters used are found in Table 1. The simulation was run with the temperature in Equation (4) set to 310 K (blue), 293 K (red), and 278 K (black).
(TIFF)
