# Supplementary figures and images for: Thixotropy and Rheopexy of Muscle Fibers Probed Using Sinusoidal Oscillations
Source: PLoS One. 2015 Apr 16;10(4):e0121726. doi: 10.1371/journal.pone.0121726 (PMC4400131; doi:10.1371/journal.pone.0121726)

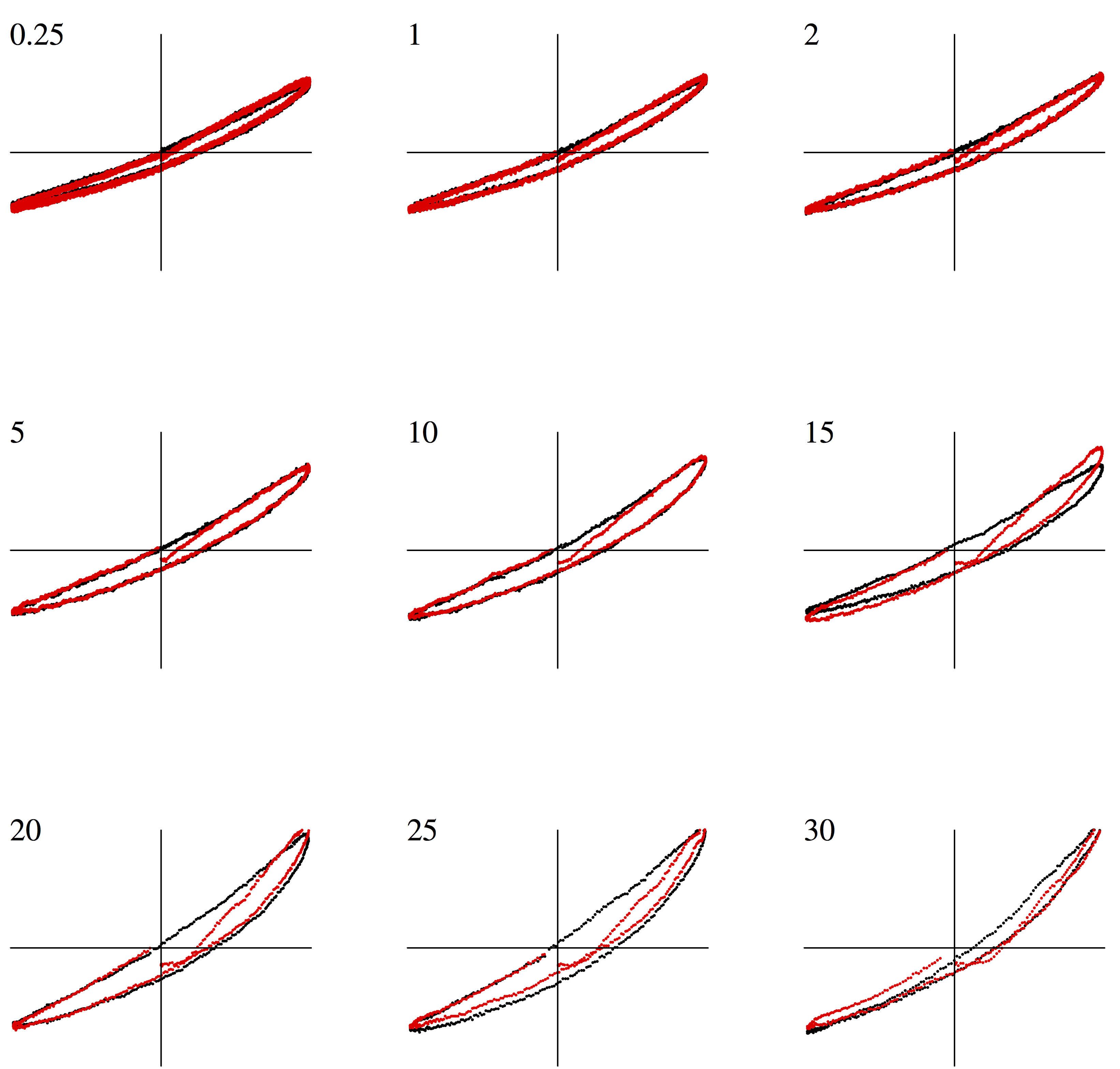

Supplement: S1 Fig — The frequency of oscillation in Hz is indicated in the top left corner of the plot. The red line is the first oscillation and the black line is the final oscillation after 20 seconds. The x-axis and y-axis are is 0.1 mm and 0.03 mN in length, respectively. The length of the muscle fiber was 2.6 mm. (TIFF) [file pone.0121726.s001.tiff]

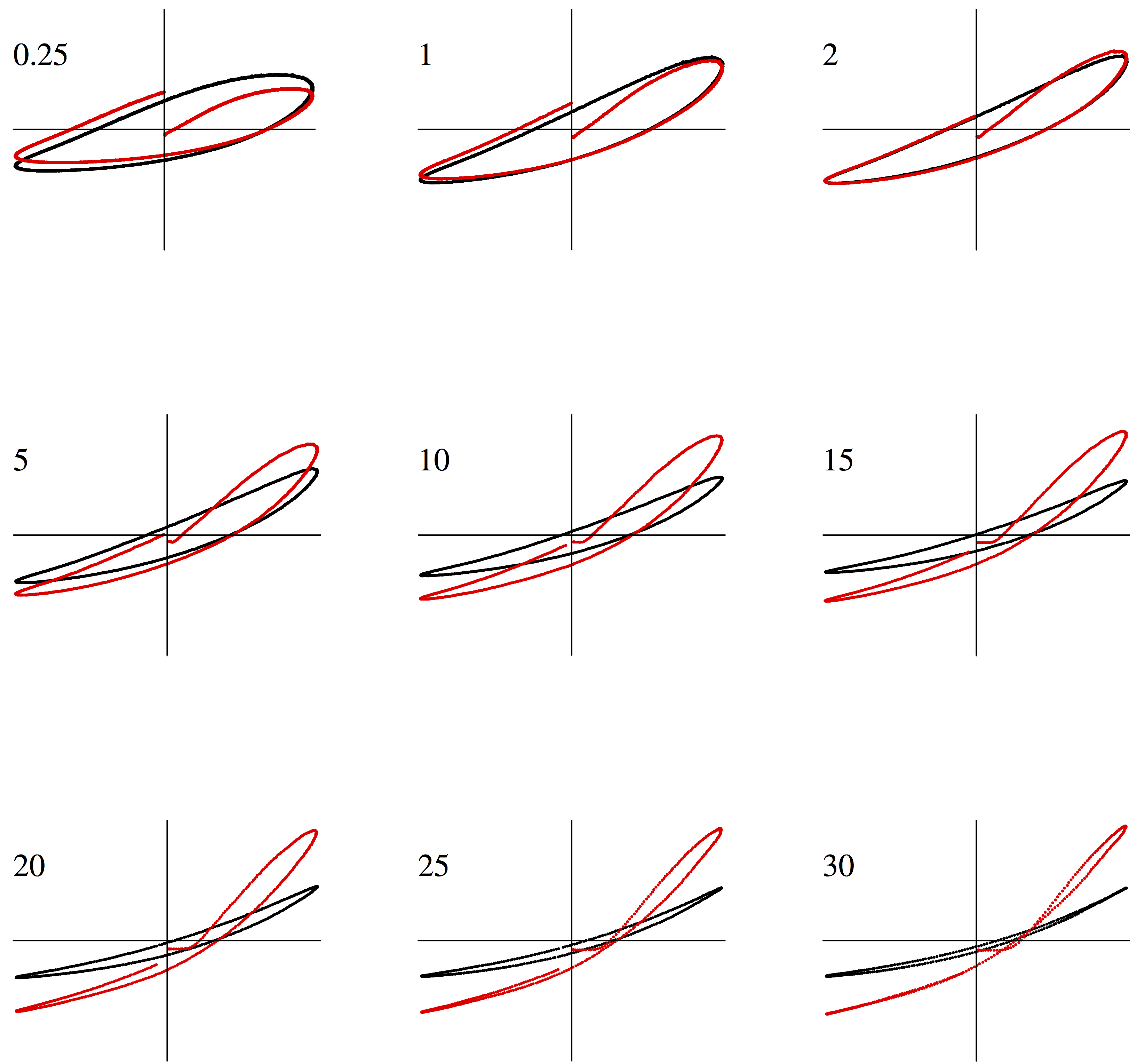

Supplement: S2 Fig — The frequency of oscillation in Hz is indicated in the top left corner of the plot. The red line is the first oscillation and the black line is the final oscillation after 20 seconds. The x-axis and y-axis are is 0.05 mm and 0.8 mN in length, respectively. The length of the muscle fiber was 2.6 mm. (TIFF) [file pone.0121726.s002.tiff]

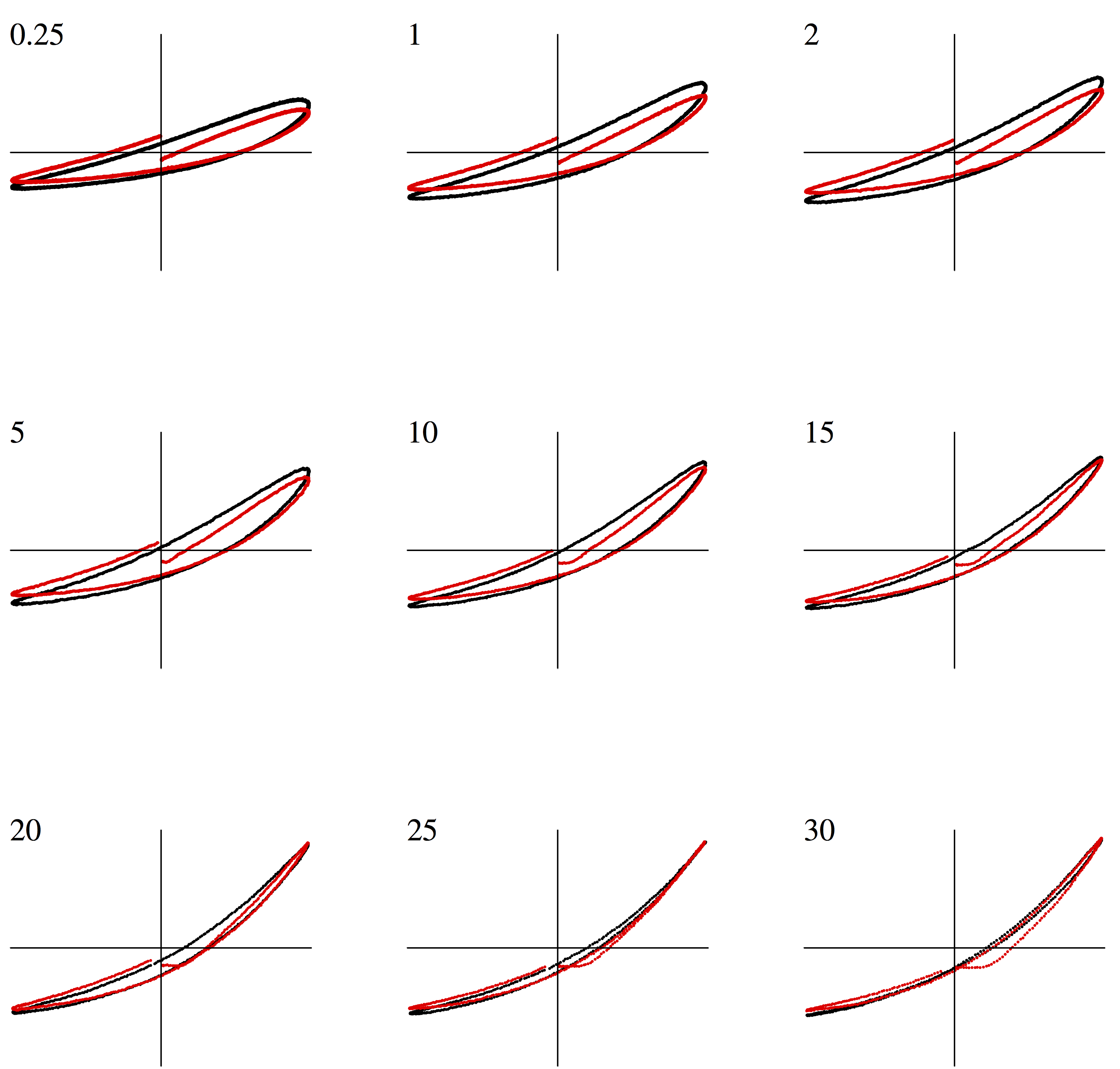

Supplement: S3 Fig — The fiber is experiencing driving sinusoidal oscillations, and the frequency of oscillation in Hz is indicated in the top left corner of the plot. The red line is the first oscillation and the black line is the final oscillation after 20 seconds. The x-axis and y-axis are is 0.05 mm and 0.11 mN in length, respectively. (TIFF) [file pone.0121726.s003.tiff]

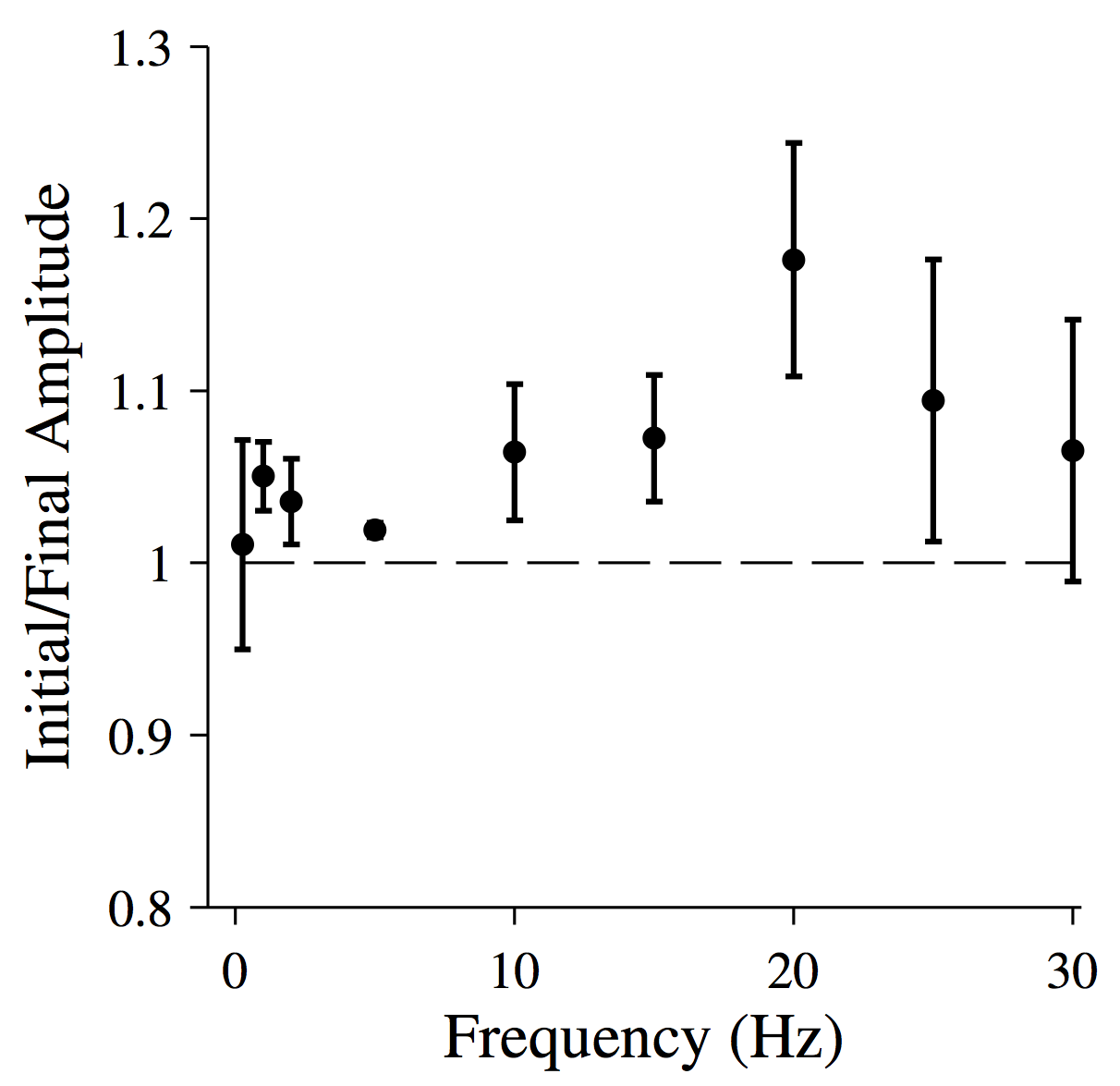

Supplement: S4 Fig — The ratio of the initial and final RMS amplitudes for relaxed muscle fibers following treatment with EDTA and blebbistatin. The total length of the oscillation was 20 seconds. All data points are (MEAN±SEM), and the number of fibers analyzed was N = 2. A one-sample t-test was used to test the null hypothesis that data collected at each frequency comes from a normal distribution with mean equal to 1. The null hypothesis could not be rejected for all data points (p>0.05). (TIFF) [file pone.0121726.s004.tiff]

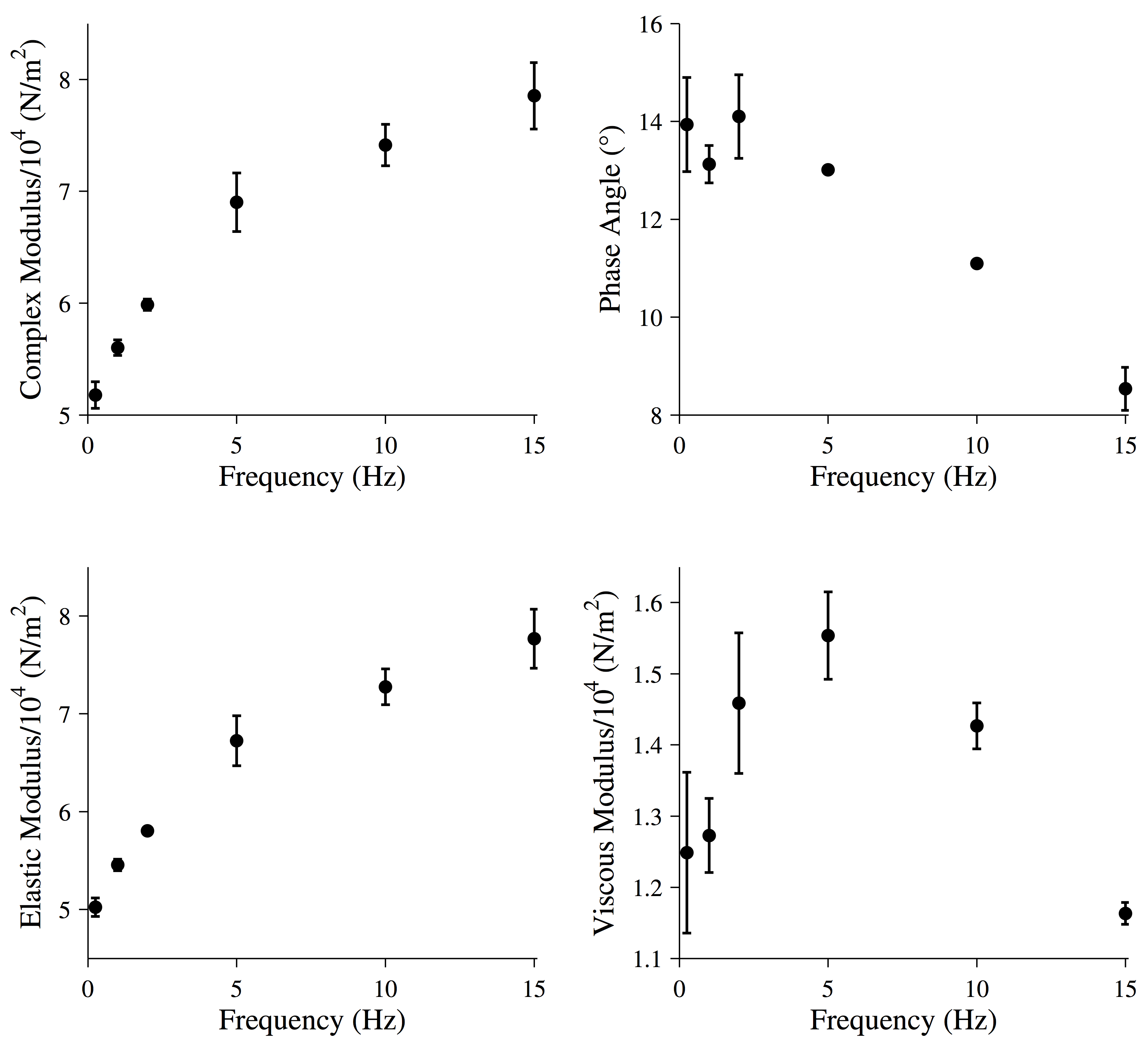

Supplement: S5 Fig — Magnitude of the complex modulus, phase, elastic modulus, and viscous modulus for relaxed muscle fibers during the final five oscillations following treatment with EDTA and blebbistatin. All data points are (MEAN±SEM), and the number of fibers analyzed was N = 2. (TIFF) [file pone.0121726.s005.tiff]

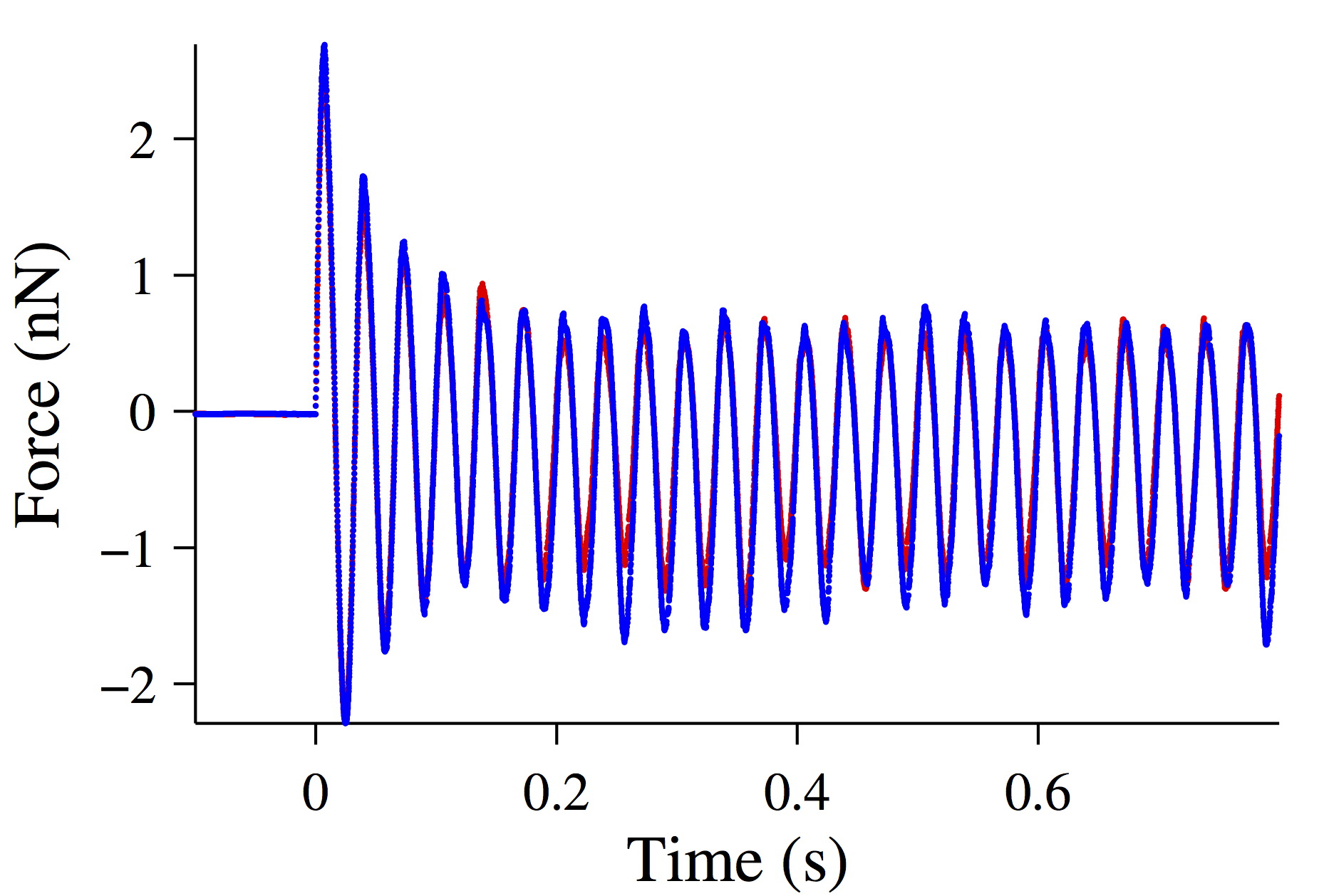

Supplement: S6 Fig — The simulation is described in detail in the text, and the parameters used are found in Table 1. The simulation was run with the temperature in Equation (4) set to 310 K (blue), 293 K (red), and 278 K (black). (TIFF) [file pone.0121726.s006.tiff]
